# The relationship between sleep quality, fatigue, and depressive symptoms in maintenance hemodialysis patients: a cross-lagged analysis

**DOI:** 10.1186/s12882-026-04780-w

**Published:** 2026-01-29

**Authors:** Hong Tan, XiaoLan Ma, HuiZe Zhang, YiPei Zhang, Li Li

**Affiliations:** 1 School of Nursing, Xinjiang Medical University, Urumqi, Xinjiang China; 2 Department of Urology, The First Affiliated Hospital of Xinjiang Medical University, Urumqi, Xinjiang 830054 China; 3 Health Care Research Center for Xinjiang Regional population, Urumqi, Xinjiang China

**Keywords:** Cross-lagged model, Sleep quality, Fatigue, Depressive symptoms, Maintenance hemodialysis

## Abstract

**Background:**

Sleep quality, fatigue, and depressive symptoms are highly prevalent and frequently co-occur as a symptom cluster in maintenance hemodialysis (MHD) patients. This cluster significantly impairs patients’ health-related quality of life and increases mortality risk. Although cross-sectional studies suggest interconnections, the temporal dynamics and interrelationships within this triad remain unclear, particularly regarding gender differences. Therefore, this study aimed to explore the temporal dynamics and longitudinal relationships among sleep quality, fatigue, and depressive symptoms in patients undergoing MHD.

**Methods:**

A longitudinal survey of 242 MHD patients in Urumqi, Xinjiang, China, was conducted to examine the interrelationships between sleep quality, fatigue, and depressive symptoms using a cross-lagged panel model. The Pittsburgh Sleep Quality Index(PSQI), Functional Assessment of Chronic Illness Therapy-Fatigue(FACIT-F), and Self-Rating Depression Scale(SDS) were used for assessment at baseline (T1, December 2024 to February 2025) and at follow-up (T2, March 2025 to May 2025).

**Results:**

Cross-lagged analyses revealed a bidirectional longitudinal relationship between lower fatigue severity (higher FACIT-F scores) and lower depressive symptoms (lower SDS scores) (β = -0.195, *P* < 0.05; β = -0.205, *P* < 0.05, respectively). Furthermore, lower baseline fatigue (higher FACIT-F scores) was longitudinally associated with better subsequent sleep quality (lower PSQI scores) (β = -0.227, *P* < 0.01). Conversely, more severe baseline depressive symptoms (higher SDS scores) predicted poorer subsequent sleep quality (higher PSQI scores) (β = 0.260, *P* < 0.01). Exploratory gender-stratified analyses revealed distinct patterns in these symptom dynamics.

**Conclusions:**

This study highlights the dynamic and reciprocal relationships among sleep quality, fatigue, and depressive symptoms in MHD patients. The reciprocal longitudinal associations between fatigue and depressive symptoms, and their links to subsequent sleep quality, underscore the importance of early identification and intervention. Interventions aimed at reducing fatigue and depressive symptoms, alongside sleep-focused strategies, may help improve sleep quality in MHD patients.

**Clinical trial number:**

Not applicable.

## Introduction

Maintenance hemodialysis (MHD) is the primary treatment for patients with end-stage renal disease (ESRD), accounting for approximately 90% of all dialysis worldwide [1]. As of December 2022, the number of individuals undergoing dialysis in China had exceeded one million [2]. Although MHD effectively controls uremia, it disrupts bodily homeostasis, leading to a substantial symptom burden that impairs health-related quality of life [3, 4]. Prominent physical symptoms include fatigue and poor sleep quality [5, 6], while psychological symptoms such as anxiety and depression are also highly prevalent [7, 8]. MHD patients frequently experience multiple co-occurring and interacting symptoms, forming identifiable symptom clusters [9]. Compared with single symptoms, symptom clusters are synergistic and reinforcing. Some of the symptoms can accumulate and have a multiplicative negative impact on patients’ quality of life and functional status, ultimately affecting dialysis outcomes and survival [10].

Sleep quality refers to an individual’s subjective evaluation of their sleep, encompassing aspects such as latency, duration, and efficiency. Sleep problems, as measured by poor sleep quality, are highly prevalent in the MHD population, frequently exceeding 50–70% [11, 12]. Causes include sympathovagal imbalance, low melatonin production, vitamin D deficiency, altered cerebral hemodynamics and hemodialysis-induced vascular stress [13]. This disruption in sleep architecture, characterized by difficulties initiating sleep, frequent awakenings, and diminished slow-wave sleep, compromises the restorative function of sleep [14] and is independently associated with reduced quality of life [15] and increased mortality [16]. In this study, sleep quality was assessed using the PSQI, which captures self-reported perceptions rather than providing a clinical diagnosis.

Fatigue, distinct from ordinary tiredness, is a pervasive, debilitating experience of persistent physical and mental exhaustion not fully alleviated by rest. It affects 70–80% of MHD patients [17], often intensifying before hemodialysis sessions and persisting throughout treatment [18]. This symptom severely restricts patients’ capacity for basic daily activities [19]and demonstrates complex bidirectional relationships with quality of life, sleep quality, and pain [20]. The transition from a fatigue-free state to fatigue is associated with a 2.18-fold higher mortality risk [21]. Therefore, for MHD patients, relief from fatigue can be as important as survival [22]. Fatigue was evaluated using the Functional Assessment of Chronic Illness Therapy–Fatigue (FACIT-F) scale, a self-report instrument measuring subjective severity, not for clinical diagnosis.

Depressive symptoms encompass singular or multiple abnormalities in emotional, cognitive, and somatic domains, such as depressed mood, diminished interest, and sleep disturbances. These symptoms may represent transient stress responses, underlying medical conditions, or other psychiatric disorders. Depression is a prevalent mental health issue among MHD patients [23]. This may stem from the persistent stress of living with a life-threatening condition, frequent hospitalizations, and dialysis regimen limitations. Untreated depression correlates with poor adherence, increased somatic symptoms, and higher mortality [24]. In this study, depressive symptoms were measured using the Self-Rating Depression Scale (SDS), which reflects patients’ subjective reports and does not equate to a clinician-defined disorder.

Cross-sectional research demonstrates significant associations between poor sleep quality, fatigue, and depressive symptoms in MHD patients [25]. Sleep disorders typically induce or exacerbate fatigue [26] because sleep deprivation disrupts the normal recovery process of bodily functions. Fatigue can also be maintained through a vicious cycle of interactions with depression and adverse behavioral patterns like sleep problems [27]. However, the temporal direction of these relationships remains unclear. Existing cross-sectional evidence cannot determine whether one symptom precedes and potentially influences the development of others over time.

Cross-lagged panel analysis (CLPA) is a key methodological approach for examining temporal sequence and potential longitudinal relationships among variables across multiple time points, providing stronger evidence for temporal precedence than correlational studies. Therefore, this study employs a CLPM to investigate the longitudinal relationships between sleep quality, fatigue, and depressive symptoms in MHD patients. The findings could inform targeted clinical interventions. However, it is crucial to note that observed associations in this design do not equate to definitive causal relationships, as they may be influenced by unmeasured confounding variables.

## Methods

### Participants and procedures

Participants were recruited via convenience sampling from two tertiary hospitals in Xinjiang, China. Specifically, 154 participants were enrolled from the First Affiliated Hospital of Xinjiang Medical University, and 88 from the People’s Hospital of Xinjiang Uygur Autonomous Region. Inclusion criteria were: (1) age ≥ 18 years; (2) having undergone MHD for > 3 months; (3) clinically stable condition, intact consciousness, and adequate communication ability; (4) provide informed consent and voluntary participation. Exclusion criteria were: (1) diagnosed cognitive impairment or psychiatric disorders; (2) comorbidities involving multiorgan failure, severe infections, or acute systemic illnesses. The study was conducted in Xinjiang, an autonomous region in northwestern China with a multi-ethnic population and distinct cultural traditions. This longitudinal study comprised two waves of data collection at a three-month interval. At the baseline (T1, conducted from December 2024 to February 2025), 334 MHD patients completed the questionnaire. Follow-up (T2, March to May 2025), involved the same questionnaire. Some participants were lost to follow-up at T2 due to health deterioration or personal reasons, resulting in 242 valid questionnaires and a response rate of 72.6%.

### Procedures

After obtaining hospital permission, study questionnaires were distributed, and anonymous surveys were conducted. All participants provided written informed consent voluntarily before participation. Participation was entirely voluntary, with the right to withdraw at any time without any benefit infringement.

This longitudinal study employed a three-phase design. Phase 1 involved recruiting participants via convenience sampling from two tertiary hospitals. Phase 2 consisted of baseline (T1) data collection via paper questionnaires administered to eligible MHD patients. During this phase, all participants consented to prospective follow-up, including explicit agreement for telephone contact. Phase 3 involved the 3-month follow-up assessment (T2) conducted via telephone interviews by trained researchers to assess longitudinal change.

This study did not collect clinical and dialysis-related variables such as comorbidities (e.g., diabetes, cardiovascular disease), dialysis adequacy (Kt/V), dialysis duration, laboratory parameters (e.g., hemoglobin, albumin), nutritional status, or use of antidepressants or hypnotics. These factors may influence the relationships examined and should be considered when interpreting the results.

### Measurement context and unassessed covariates

Both assessments in this study aimed to capture patients’ daily symptom experiences, but contextual differences and information gaps existed: The baseline assessment (T1) was conducted face-to-face in a nephrology ward setting; the follow-up assessment (T2) was completed via telephone. Neither assessment recorded whether it occurred on a hemodialysis day nor distinguished between daytime and nighttime dialysis sessions. Furthermore, the study did not collect behavioral factors that may significantly moderate sleep and fatigue, such as daytime napping frequency and duration, daily physical activity levels, or typical patterns of fatigue fluctuation between dialysis sessions. The absence of these variables may introduce additional variability into symptom scores and affect the precise inference of longitudinal associations.

### Measures

#### The Pittsburgh Sleep Quality Index (PSQI)

PSQI was developed by Buysse [28] as a validated psychometric instrument containing 18 items in 7 dimensions (subjective sleep quality, sleep latency, sleep duration, sleep efficiency, sleep disturbances, daytime dysfunction, and use of sleep medication). Each dimension is scored 0–3, with total scores ranging from 0 to 21. Higher total scores indicate poorer sleep quality, with a clinical cutoff > 7 distinguishing individuals with sleep disorders [29]. The scale demonstrated Cronbach’s α of 0.828 at T1 and 0.826 at T2.

#### Self-Rating Depression Scale (SDS)

SDS was developed by Zung [30], is a unidimensional scale containing 20 items rated on a 4-point Likert scale (1=“never” to 4=“always”). Standardized scores are derived by multiplying raw totals by 1.25. Higher scores indicate more severe depressive symptoms. Clinical thresholds are: ≤53(normal), 53–62(mild), 63–72(moderate), and ≥ 72(severe). The scale demonstrated Cronbach’s α of 0.949 at T1 and 0.928 at T2.

#### Functional Assessment of Chronic Illness Therapy-Fatigue (FACIT-F)

FACIT-F was developed by Yellen [31], was originally designed to assess fatigue in anemic cancer patients; it was later extended to chronic disease populations. This single-dimensional scale contains 13 items (total score range: 0–52). Higher scores indicate lower fatigue severity, with a score < 36 serving as the clinical cutoff for significant fatigue [32]. Wang et al. [33] validated the Chinese version in MHD patients, confirming its reliability and validity. The scale demonstrated Cronbach’s α of 0.905 at T1 and 0.906 at T2.

All measures relied on self-reported data. Despite efforts to standardize procedures and ensure confidentiality, common method bias might still be present. Harman’s single-factor test was applied within the longitudinal follow-up program using an unrotated factor solution to assess the overall variance explained by common factors.

### Data analysis

Statistical analyses were performed using SPSS 23.0 and R 4.4.2. Common method bias testing, descriptive statistics, and correlation analyses were conducted in SPSS 23.0. The CLPM was constructed in R 4.4.2 to examine the longitudinal relationships.

### Ethical considerations

The study was approved by the hospital ethics committee (K202412-77). All procedures followed the Declaration of Helsinki. Patients were informed about the research aims, procedures, and their unconditional right to withdraw without detriment. Researchers guaranteed their anonymity and confidentiality, ensuring that no identifying factors or information about participants were disclosed throughout the study and publication process.

## Results

### Common method bias test

Common method bias was assessed using Harman’s single-factor test. An unrotated exploratory factor analysis (EFA) was performed on all items at both time points. For T1 data, the EFA extracted 8 factors with eigenvalue > 1, of which the first factor cumulatively explained 38.31% of the total variance. For T2 data, 8 factors emerged (eigenvalue > 1), with the first factor accounting for 32.71% of the total variance. As the variance explained by the first factor was below 40% at both T1 and T2, significant common method bias was not detected.

### Descriptive and correlation analysis

The proportion of patients meeting clinical cutoffs for each symptom at both time points is presented in Table 1. At baseline, over half reported poor sleep quality (59.5%) and clinically relevant depressive symptoms (51.7%). Descriptive statistics and bivariate correlations for sleep quality, fatigue, and depressive symptoms are summarized in Table 2. Since the Shapiro-Wilk test indicated significant departures from normality for all continuous variables, data are presented as median (IQR). At T1, significant negative correlations were observed between PSQI scores and FACIT-F scores, and between FACIT-F scores and SDS scores, while a positive correlation was found between PSQI scores and SDS scores. These patterns remained consistent at T2. Notably, significant negative correlations were found between T1 PSQI scores and T2 FACIT-F scores, and between T1 FACIT-F scores and T2 SDS scores. Conversely, a significant positive correlation was observed between T1 SDS scores and T2 PSQI scores. These findings indicate complex temporal interrelationships between sleep quality, fatigue, and depressive symptoms.

**Table 1:** Prevalence of clinically significant symptoms at T1 and T2 (*N* = 242)

Symptom / Scale (Cutoff Score)	T1, *n* (%)	T2, *n* (%)
Poor Sleep Quality (PSQI > 7)	144(59.5%)	131(54.1%)
Significant Fatigue (FACIT-F < 36)	102(42.1%)	82(33.9%)
Clinically Relevant Depressive Symptoms (SDS ≥ 53)	125(51.7%)	57(23.6%)
Mild Depressive Symptoms (SDS: 53–62)	46(19.0%)	24(9.9%)
Moderate Depressive Symptoms (SDS: 63–72)	44(18.2%)	19(7.9%)
Severe Depressive Symptoms (SDS: ≥ 72)	35(14.5%)	14(5.8%)

Note. Data show the proportion of paired samples meeting these thresholds at baseline (T1) and 3-month follow-up (T2)

**Table 2:** Descriptive statistics and bivariate correlations for study variables at T1 and T2 (*N* = 242)

Variable	Time Point	Median (IQR)	1		2	3		4		5	6
1. PSQI	T1	10.00 (5.00, 15.00)	1								
2. FACIT-F	T1	39.50 (25.00, 46.00)	-0.442^**^		1						
3. SDS	T1	53.75 (43.75, 68.75)	0.545^**^		-0.697^**^	1					
4. PSQI	T2	8.00 (5.00, 14.00)	0.497^**^		-0.511^**^	0.531^**^		1			
5. FACIT-F	T2	40.00 (32.00, 47.00)	-0.288^**^		0.396^**^	-0.408^**^		-0.435^**^		1	
6. SDS	T2	45.00 (37.50, 52.50)	0.314^**^		-0.404^**^	0.451^**^		0.409^**^		-0.542^**^	1

Note: PSQI, higher scores indicate poorer sleep quality; FACIT-F, higher scores indicate lower fatigue severity; SDS, higher scores indicate more severe depressive symptoms; **P < 0.01

### Longitudinal changes in symptom scores

To examine whether there were significant changes in sleep quality, fatigue, and depressive symptoms between T1 and T2, paired samples comparisons were conducted. Given the non-normal distribution of the data (as indicated by the Shapiro-Wilk test, *P* < 0.05 ), the Wilcoxon signed-rank test was used. The results showed that sleep quality (PSQI) did not change significantly (Z = -1.317, *P* = 0.188). In contrast, both fatigue and depressive symptoms changed significantly: fatigue scores (FACIT-F) decreased (Z = -3.368, *P* = 0.001), indicating an increase in fatigue severity, while depressive symptoms (SDS) also decreased significantly (Z = -9.030, *P* < 0.001). These findings indicate a differential change pattern among symptoms over the three months, with depressive symptoms showing the most pronounced improvement.

### Cross-lagged panel model

**Fig. 1:**
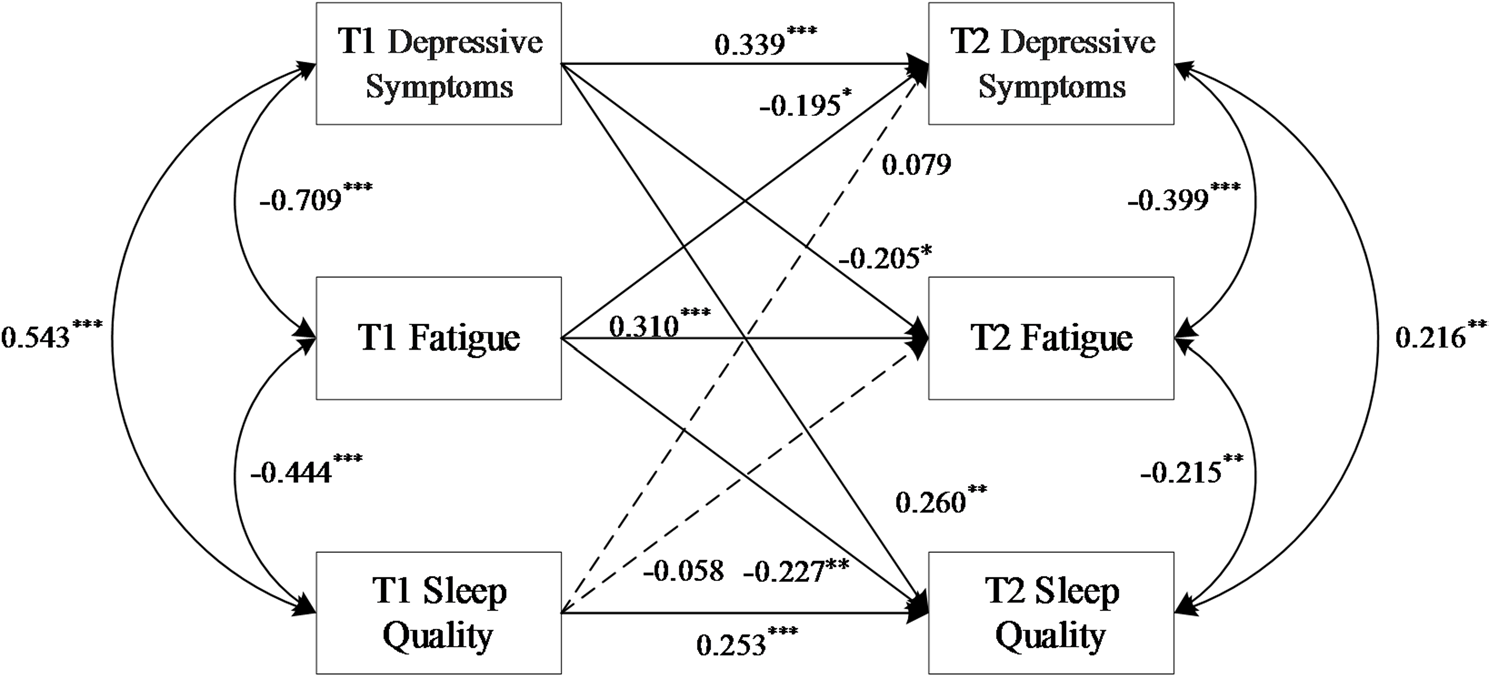
Cross-lagged panel model of associations between sleep quality, fatigue, and depressive symptoms. Path coefficients are standardized estimates (β). Solid arrows indicate statistically significant paths (*P* < 0.05); dashed arrows represent non-significant paths. Covariates (gender, ethnicity, and age) were included but are omitted for clarity. ***P < 0.001, **P < 0.01, *P < 0.05

Figure 1 presents the results of the CLPM for sleep quality, fatigue, and depressive symptoms. As a saturated model, fit statistics were not applicable. Analyses revealed four significant cross-lagged paths. A bidirectional longitudinal association was observed between lower fatigue (higher FACIT-F scores) and lower depressive symptoms (lower SDS scores) (T1 FACIT-F scores→ T2 SDS scores: *β* = -0.195; T1 SDS scores → T2 FACIT-F scores: *β* = -0.205; both *P* < 0.05). In addition, lower baseline fatigue (higher FACIT-F scores) was longitudinally associated with better subsequent sleep quality (lower PSQI scores) (*β* = -0.227, *P* < 0.01). Conversely, more severe baseline depressive symptoms (higher SDS scores) predicted poorer subsequent sleep quality (higher PSQI scores) (*β* = 0.260, *P* < 0.01). In contrast, baseline sleep quality did not significantly predict follow-up depressive symptoms or fatigue (both *P* > 0.05).

### Cross-lagged panel model for male and female

As an exploratory analysis, we examined the cross-lagged relationships separately by gender. Given the established clinical and epidemiological distinctions in fatigue, sleep quality, and depressive symptoms across genders, we present the model parameters for males and females descriptively to inform future research and potential intervention strategies.

**Fig. 2:**
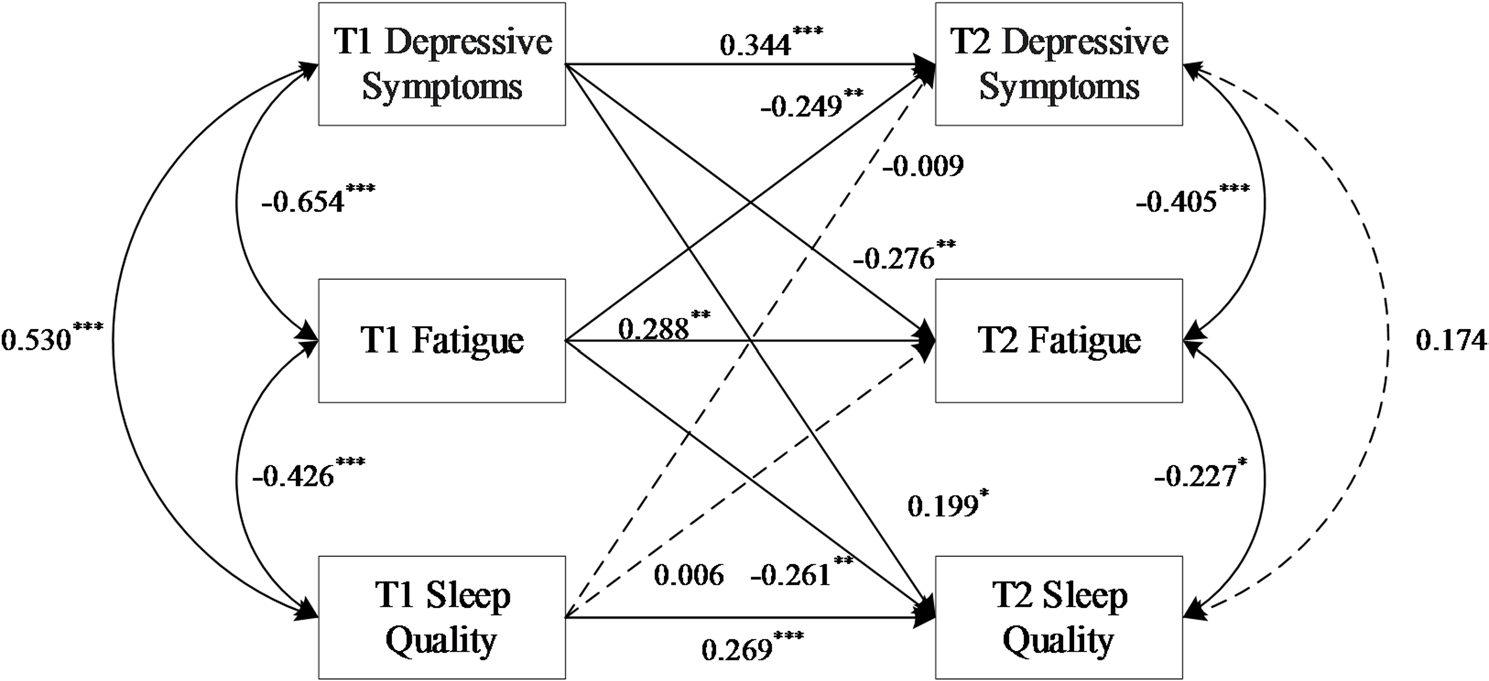
Cross-lagged panel model of associations between sleep quality, fatigue, and depressive symptoms for males. Path coefficients are standardized estimates (β), with solid arrows indicating statistically significant paths (*P* < 0.05) and dashed arrows representing non-significant paths. Covariates (ethnicity and age) were included but are omitted from the diagram for clarity. ***P < 0.001, **P < 0.01, *P < 0.05

Figure 2 presents the CLPM results for males. The model exhibited a good fit: χ²/df = 1.655, GFI = 0.990, TLI = 0.955, RMSEA = 0.063, SRMR = 0.058. Significant cross-lagged paths revealed a bidirectional longitudinal association between lower fatigue (higher FACIT-F scores ) and lower depressive symptoms (lower SDS scores) (T1 FACIT-F scores → T2 SDS scores: *β* = -0.249; T1 SDS scores → T2 FACIT-F scores: *β* = -0.276; both *P* < 0.01). Additionally, lower baseline fatigue (higher FACIT-F scores) predicted better subsequent sleep quality (lower PSQI scores) (*β* = -0.261, *P* < 0.01). Conversely, more severe baseline depressive symptoms (higher SDS scores) predicted poorer subsequent sleep quality (higher PSQI scores) (*β* = 0.199, *P* < 0.01). In contrast, baseline sleep quality did not significantly predict follow-up depressive symptoms or fatigue (both *P* > 0.05). The concurrent association between depressive symptoms and sleep quality at T2 was also nonsignificant (*β* = 0.174, *P* > 0.05).

**Fig. 3:**
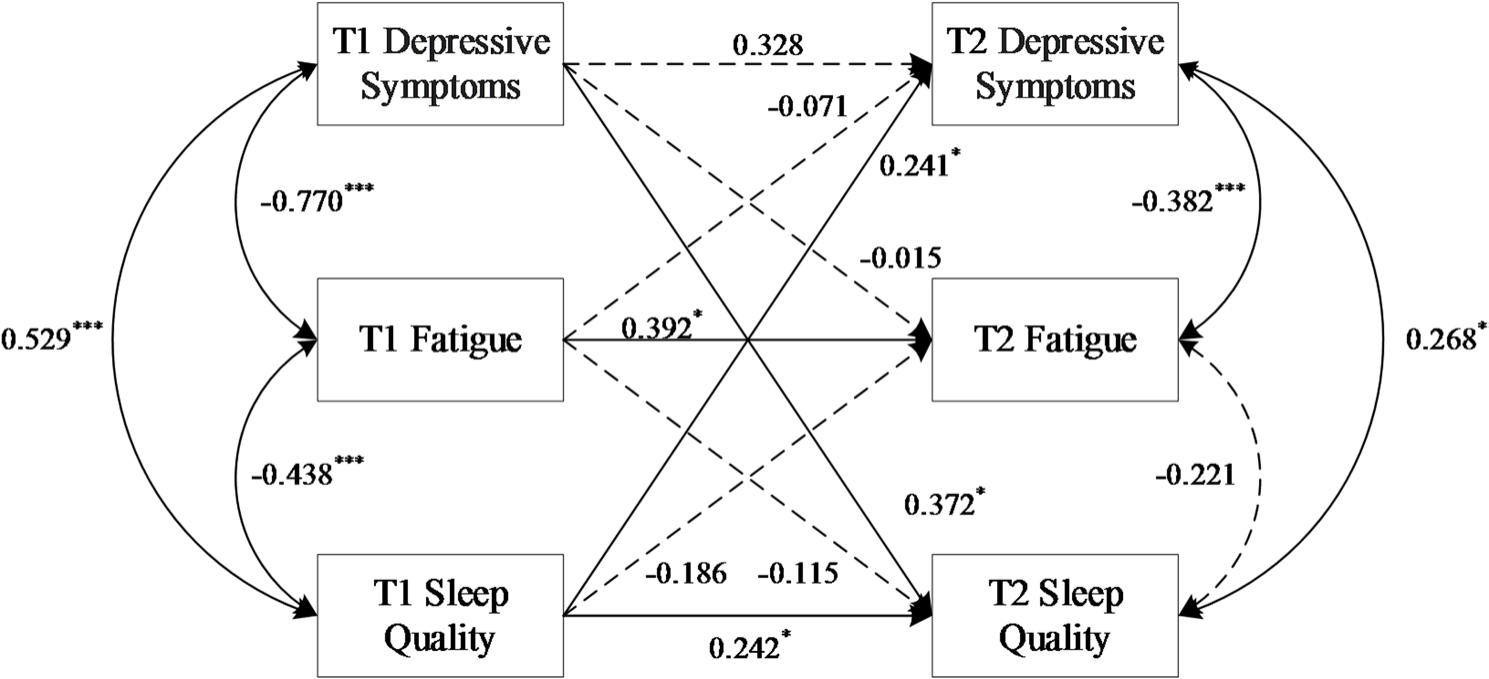
Cross-lagged panel model of associations between sleep quality, fatigue, and depressive symptoms for females. Path coefficients are standardized estimates (β), with solid arrows indicating statistically significant paths (*P* < 0.05) and dashed arrows representing non-significant paths. Covariates (ethnicity and age) were included but are omitted from the diagram for clarity. ***P < 0.001, **P < 0.01, *P < 0.05

Figure 3 presents the CLPM results for females. The model exhibited an acceptable fit: χ2/df = 1.642, GFI = 0.980, TLI = 0.909, RMSEA = 0.093, SRMR = 0.076. Key findings revealed a bidirectional longitudinal relationship wherein more severe depressive symptoms (higher SDS scores) predicted poorer subsequent sleep quality (higher PSQI scores), and vice versa (T1 SDS scores → T2 PSQI scores: *β* = 0.372; T1 PSQI scores → T2 SDS scores: *β* = 0.241; both *P* < 0.05). Conversely, cross-lagged paths from depressive symptoms to fatigue and from fatigue to sleep quality were non-significant (both *P* > 0.05). The autoregressive path for depressive symptoms and the concurrent association between fatigue and sleep quality at T2 were also non-significant (all *P* > 0.05).

## Discussion

### Reciprocal relationships and sex differences among sleep quality, fatigue, and depressive symptoms

This study investigated the reciprocal associations among sleep quality, fatigue, and depressive symptoms from a longitudinal perspective. To this end, we utilized data from Xinjiang, China, applying a cross-lagged panel model to elucidate the temporal dynamics of these symptoms in MHD patients. We observed bidirectional longitudinal associations between fatigue and depressive symptoms across both time points. Furthermore, Lower fatigue severity and milder depressive symptoms at baseline were longitudinally associated with better subsequent sleep quality. Notably, exploratory sex-stratified analyses revealed differing patterns in the symptom pathways: males exhibited bidirectional associations between fatigue and depressive symptoms, and both symptoms showed associations with subsequent sleep quality, whereas females showed reciprocal associations between depressive symptoms and sleep quality. Although these findings illuminate complex symptom interactions, future research should investigate temporal gender effects and the underlying biological mechanisms. Clinically, results underscore the necessity for integrated screening protocols targeting this symptom triad, with early intervention potentially with early intervention potentially mitigating the progression of this symptom cluster in MHD populations.

### Reciprocal longitudinal associations between fatigue and depressive symptoms

Our investigation is motivated by cross-sectional evidence establishing the significant co-occurrence and clinical impact of these symptoms. For instance, Matsunaga [34] demonstrated that overlapping symptoms of depression, apathy, and sleep disturbances were independently associated with reduced activities of daily living in hemodialysis patients, highlighting the functional consequences of this symptom cluster. Contrasting prior cross-sectional evidence, this longitudinal study identified reciprocal temporal associations between fatigue and depressive symptoms in MHD patients from T1 to T2, suggesting they are dynamically linked over time. This bidirectional relationship may be mediated by inflammatory pathways [35, 36]. Specifically, depression activates the hypothalamic-pituitary-adrenal axis and sympathetic nervous system, leading to increased levels of pro-inflammatory cytokines, which in turn are associated with fatigue [37]. Concurrently, fatigue-driven physical inactivity [38] reduces the production of actin (Irisin) [39], diminishing anti-inflammatory responses while promoting inflammasome activation [40]. This pattern of reciprocal longitudinal association is consistent with a potential self-perpetuating cycle. The clinical implications of these findings are that integrated management strategies targeting both fatigue and depressive symptoms could be considered. Early intervention for either symptom might help to reduce the overall symptom burden. Therefore, these findings suggest a need to move beyond traditional single-system management towards integrated, cross-system intervention strategies to improve the quality of life of MHD patients by addressing both depressive symptoms and fatigue concurrently at an early stage.

### Longitudinal associations of depressive symptoms and fatigue with subsequent sleep quality

Longitudinal cross-lagged analyses revealed two unidirectional predictive pathways. Lower fatigue at T1 (higher FACIT-F scores) significantly predicted better sleep quality at T2 (lower PSQI scores), with no significant pathway in the reverse direction. Conversely, more severe depressive symptoms at T1 (higher SDS scores) were associated with poorer subsequent sleep quality, again without a significant reverse effect from sleep to depressive symptoms. This asymmetry may stem from two interconnected mechanisms: First, depressive symptoms and fatigue directly disrupt sleep preparatory behaviors and circadian regulation [41]. Second, MHD patients face unique environmental stressors (frequent dialysis schedules, dietary restrictions, and activity limitations) [42], which in themselves are important triggers of depression and fatigue. Within this framework, poor sleep quality may follow these symptoms rather than act as an independent driver. Although extant literature implicates sleep disorders in exacerbating fatigue and depression [26, 43], such effects were undetected here. This may be due to an insufficient follow-up interval to capture the potential long-term cumulative effects of sleep disturbance on depression or fatigue. Clinically, the longitudinal link from fatigue to sleep suggests that changes in fatigue levels may be a relevant marker for future changes in sleep quality. Specifically, a reduction in fatigue could signal an opportunity for sleep improvement, whereas persistent or worsening fatigue might indicate a risk for ongoing sleep disturbance. Similarly, depressive symptoms could be considered a potential marker for impending sleep dysfunction in MHD patients, warranting increased clinical attention. Routine sleep assessments with targeted interventions are recommended when these symptoms emerge. Conversely, while sleep optimization improves quality of life, our data indicate that improving sleep quality alone may not be sufficient to effectively alleviate already existing core symptoms of depression and fatigue, which calls for a more comprehensive clinical management program that directly targets the underlying etiology of depression and fatigue.

It is noteworthy that the association between sleep quality and subsequent depressive symptoms and fatigue was not significant in our model, which may be partly attributed to the short follow-up interval. The impact of sleep quality may accumulate over longer periods or operate through indirect pathways that were not captured within the three-month window. Therefore, future longitudinal studies with extended follow-up durations are needed to clarify the potential long-term role of sleep quality within this symptom network.

### Exploring potential mechanisms for sex-specific patterns

The observed sex-specific pathways in our CLPM are characterized by bidirectional associations between fatigue and depressive symptoms in males and bidirectional associations between depressive symptoms and sleep quality in females. These patterns may arise from an interplay of biological and psychosocial factors.

Hormonal fluctuations and gender-specific physiological responses triggered by chronic diseases may be contributing factors. For women, estrogen plays a central role in regulating circadian rhythms, neurotransmitter balance, and inflammatory pathways—mechanisms closely linked to sleep [44] and mood regulation [45]. Furthermore, physiological stress associated with MHD may exacerbate estrogen dysregulation in women, which could underlie the observed bidirectional temporal link between depressive symptoms and sleep quality. In contrast, males exhibit higher levels of testosterone, a hormone that regulates energy metabolism [46]. Due to renal dysfunction, MHD patients frequently experience testosterone deficiency [47]. Testicular insufficiency is closely linked to the nervous system, leading to fatigue and depressive symptoms in patients [48]. This provides a plausible biological account for the bidirectional association between fatigue and depressive symptoms observed in this study.

Gender differences in coping strategies may also shape variations in the associations between variables. Research indicates that women are more prone to depressive symptoms and ruminative thinking, while men tend to somatize psychological stress into physical symptoms [49]. In the context of depressive sleep disorders, female patients may perceive emotional fluctuations more acutely, leading to stronger bidirectional effects between depressive symptoms and sleep disturbances [50]. In contrast, men may suppress emotional expression [51] but report fatigue more prominently, as physical exhaustion aligns with the “resilient” trait expected by traditional gender norms. This difference in symptom focus could contribute to the maintenance of a bidirectional linkage: unacknowledged depression manifests as fatigue, which in turn limits physical activity and social engagement, thereby exacerbating depression.

### Clinical implications

This study highlights critical clinical implications for managing sleep quality in MHD patients. Our findings show that reduced fatigue is longitudinally associated with better subsequent sleep quality. Conversely, more severe depressive symptoms show a longitudinal association with poorer sleep quality. This suggests that while standard sleep hygiene advice remains relevant, incorporating strategies that specifically aim to reduce fatigue and alleviate depressive symptoms, alongside sleep-focused interventions, might lead to more comprehensive improvements in patient well-being. We therefore propose a structured, multi-component intervention approach. First, routine screening for depression and fatigue should be integrated into sleep quality management protocols for MHD patients, utilizing validated scales to enable early identification of these conditions. For patients with clinically significant symptoms, priority should be given to evidence-based interventions such as cognitive-behavioral therapy for depression and individualized physical activity programs for fatigue. Furthermore, intervention plans should be tailored to individual needs, including those based on gender. Our gender-stratified results suggest different intervention priorities: interventions that concurrently target the reduction of fatigue and depression appear salient for male patients, whereas addressing the link between depression and sleep may be particularly relevant for females. In summary, improving sleep in MHD patients requires an integrated strategy that concurrently addresses depression and fatigue, moving beyond a sole focus on sleep hygiene.

## Limitations

The study has some limitations. First, the generalizability of findings may be constrained by regional context. All participants were recruited using convenience sampling from only two tertiary hospitals in Urumqi, Xinjiang, a region with distinct ethnic, cultural, and socioeconomic characteristics. Second, data relied exclusively on self-report measures. Although Harman’s single-factor test did not indicate severe common method bias, its inherent limitations preclude ruling out such bias entirely. Third, we did not collect important clinical and dialysis-related covariates such as comorbidities, dialysis adequacy, dialysis duration, laboratory parameters, nutritional status, and medication use, which may confound the observed associations. Fourth, the three-month follow-up may be too short to capture longer-term symptom dynamics or delayed effects of sleep quality, which could explain its non-significant cross-lagged paths. Additionally, attrition bias may have influenced results, as the 92 participants lost to follow-up might have had more severe symptoms. Thus, the findings represent temporal associations rather than confirmed causal relationships and should be interpreted with caution. Finally, we relied on continuous symptom measurement and did not conduct subgroup analyses based on symptom severity. Consequently, our findings reflect population-level average associations and may not capture differential dynamics across severity subgroups.

## Conclusions

This longitudinal study revealed distinct symptom trajectories over three months: sleep quality remained stable, fatigue increased in severity, while depressive symptoms showed significant improvement. Cross-lagged analyses indicated reciprocal longitudinal associations between fatigue and depressive symptoms. Furthermore, lower fatigue was longitudinally associated with better subsequent sleep quality, whereas more severe baseline depressive symptoms were associated with poorer subsequent sleep quality. In contrast, baseline sleep quality did not significantly predict later fatigue or depressive symptoms. Exploratory analyses by gender suggested differing patterns in symptom dynamics. These findings highlight the interconnected nature of this symptom cluster in MHD patients and suggest that management strategies concurrently addressing fatigue and depressive symptoms may contribute to improved sleep outcomes.

## Data Availability

The data that support the findings of this study are available from the corresponding author Dr. Li Li upon reasonable request.

## Abbreviations

MHD: Maintenance hemodialysis
CLPM: Cross-lagged panel model
ESRD: End-stage renal disease
FACIT-F: Functional Assessment of Chronic Illness Therapy–Fatigue
SDS: Self-Rating Depression Scale
PSQI: Pittsburgh Sleep Quality Index
